# Associations of current Schistosoma mansoni infections with hepatosplenic organometry: a community-based cross-sectional study within the SchistoTrack cohort in rural Uganda

**DOI:** 10.1136/bmjph-2026-005503

**Published:** 2026-11-09

**Authors:** Kayla Seggelke, Max Melchior Lang, Betty Nabatte, Victor Anguajibi, Benjamin Ntegeka, Tymothy Mugume, Simon Mpooya, Narcis B Kabatereine, Adrian D Smith, Goylette F Chami

**Affiliations:** 1 Nuffield Department of Population Health, Big Data Institute, University of Oxford, Oxford, UK; 2 Division of Vector-Borne and Neglected Tropical Diseases, Republic of Uganda Ministry of Health, Kampala, Uganda; 3 Ugandan Institute of Allied Health & Management Sciences, Kampala, Uganda; 4 Nuffield Department of Population Health, University of Oxford, Oxford, UK

**Keywords:** Public Health, Epidemiology, Endemic Diseases

## Abstract

**Introduction:**

*Schistosoma mansoni* is a leading cause of hepatosplenic disease in sub-Saharan Africa. Yet, associations with current *S. mansoni* infections and hepatosplenic organometry remain unclear in the context of widespread mass drug administration and co-endemic infections.

**Methods:**

From January to February 2024, we conducted a community-based, cross-sectional study nested within the SchistoTrack cohort in three districts of Uganda. Liver and spleen dimensions were assessed via point-of-care B-mode ultrasound for 3121 individuals. Organ dimensions were classified using the SDs from height-standardised internal reference values derived from an infection-free population. Multinomial logistic regressions were run for children (5–17 years) and adults (18+ years) separately. Population attributable fractions (PAFs) were used to estimate the proportion of abnormal organometry statistically attributable to each infection. Key exposures were *S. mansoni*, malaria, HIV and hepatitis B virus (HBV) alongside controlling for a comprehensive set of social, biomedical and other covariates.

**Results:**

Moderate-to-severe splenic enlargement was observed in 29.1% (438/1507) of children and 23.3% (376/1614) of adults. Among adults, 20.9% (337/1614) had left liver lobe enlargement and 18.8% (303/1614) had right liver lobe shrinkage. In children, severe splenic enlargement was statistically attributable to malaria (PAF 46.7%; relative risk ratio (RRR) 3.96, 95% CI 2.64 to 5.92) and continuous *S. mansoni* infection intensity (ln eggs per gram+1; PAF 23.6%; RRR 1.12, 95% CI 1.04 to 1.20). In adults, *S. mansoni* intensity was positively associated with moderate left liver lobe enlargement (PAF 12.4%; RRR 1.11, 95% CI 1.04 to 1.18). In adults, HIV was associated with severe left liver lobe shrinkage (RRR 4.50, 95% CI 1.19 to 17.00) and severe splenomegaly (RRR 3.62, 95% CI 1.58 to 8.33), while HBV was associated only with severe left liver lobe shrinkage (RRR 2.54, 95% CI 1.07 to 6.03).

**Conclusions:**

Current *S. mansoni* infection intensity remains associated with splenomegaly in children, despite controlling for concurrent malaria positivity and with hepatomegaly in adults despite HIV and HBV associations.

**WHAT IS ALREADY KNOWN ON THIS TOPIC**

- Hepatosplenic morbidity is a known complication of *Schistosoma mansoni* infection, but the correlation between current infection status and specific organ abnormalities remains uncertain in settings with widespread praziquantel mass drug administration and co-endemic infections like malaria, HIV and hepatitis B virus (HBV).

**WHAT THIS STUDY ADDS**

- We demonstrated that current *S. mansoni* infection intensity remains positively associated with paediatric splenomegaly, independent of concurrent malaria, and with adult hepatomegaly. HIV and HBV co-infections contributed to the burden of severe liver abnormalities in adults. Notably, we provide an update to organometry reference tables for schistosomiasis as WHO Niamey Protocol reference values systematically misclassified local organ morbidity.

**HOW THIS STUDY MIGHT AFFECT RESEARCH, PRACTICE OR POLICY**

- We challenged current assumptions regarding morbidity surveillance, suggesting that clinical guidelines may need to retain paediatric splenomegaly assessments in malaria-endemic areas while also accounting for viral co-infections in adults. The height-standardised organometry reference tables developed here can offer a more accurate framework for routine clinical evaluation than the existing WHO Niamey reference tables.

## Introduction

 Schistosomiasis is endemic in at least 41 countries in sub-Saharan Africa (SSA), where an estimated 237 million people require treatment for current schistosome infections.^1^ Hepatosplenic schistosomiasis caused by *Schistosoma mansoni* is common in SSA. For example, the prevalence of hepatosplenic schistosomiasis has been estimated to be as high as 55% in children and adults in rural communities in Uganda.^2^ The eggs produced by the female parasitic flukes induce granulomatous inflammatory responses that, if uncontrolled by the host, may lead to periportal fibrosis (PPF), including portal vasculature restructuring. Changes in spleen and liver sizes often precede or accompany PPF.^2,3^ It remains unknown whether current *S. mansoni* infection correlates with hepatosplenic organometry, given the widespread availability of praziquantel through mass drug administration (MDA) and co-endemic infections of malaria, HIV and hepatitis B virus (HBV).

The assessment of organometry, mostly focused on hepatosplenomegaly, has been used for early indications of disease in children due to the heaviest burden of infection being concentrated in this group.^4–7^ However, recently, it has been shown that there is a disconnect between more severe hepatosplenic outcomes, in particular PPF that accompanies easily observed changes in organometry, and current intestinal schistosome infection.^3^ The highest burden of PPF has been shown to be in adults, supporting the need for additional studies within this age group that also rely on community-based sampling rather than school-based sampling.^3^ It is assumed that schistosomiasis rarely causes cirrhosis or cirrhosis-like outcomes, including shrunken livers,^8^ yet few studies have examined these severe outcomes.

There is no agreement as to the direction and significance of organometry and current schistosome infections within the context of routine praziquantel treatment or MDA. For instance, while some studies report significant associations between *S. mansoni* and hepatosplenomegaly,^4,7^ others have found no significant difference in hepatosplenomegaly based on infection status.^4,5,9,10^ Detectable morbidity may depend on infection intensity, as several studies note that hepatosplenomegaly was only significantly associated with *S. mansoni* in individuals with heavy infections.^11–13^ Yet, for related outcomes of PPF, there is no association of current intestinal schistosome infections and PPF within the context of routine MDA.^14^ The lack of morbidity management guidelines for schistosomiasis, coupled with the inability of MDA to resolve schistosomiasis-associated morbidities, highlights the need to revisit the use of routine organometry for assessing schistosomiasis-related morbidity in endemic countries.

Schistosomiasis often is co-endemic with malaria, HIV and HBV in rural SSA.^15–18^ Malaria and schistosome co-infections are most often studied with respect to splenomegaly only.^7,19,20^ Yet, there is a disagreement as to whether splenomegaly should be assessed for schistosomiasis in areas endemic with malaria. The WHO protocol for assessing schistosomiasis morbidity (Niamey Protocol) recommends against assessing splenomegaly in malaria endemic areas with the assumption that malaria is the sole cause.^21^ Yet, it has been shown that malaria infection facilitates schistosome-initiated splenomegaly.^6^

HIV and HBV have been shown to independently correlate with outcomes related to cirrhosis, and as such, shrunken livers,^22,23^ whether schistosome infections independently contribute to this outcome or exacerbate HIV and HBV pathology remains to be explored. To our knowledge, no studies examine both schistosome and either HIV or HBV infections and associated organometry. A recent meta-analysis found that current schistosome infection is not associated with PPF in the context of MDA, suggesting that current infection status may not be the best proxy for cumulative morbidity or severe fibrosis/cirrhosis risk,^14^ though HIV infection has been shown to be linked to PPF.^2,3^

We conducted a study of organometry using point-of-care B-mode ultrasound on 3121 individuals aged 5–90 years in the SchistoTrack cohort in rural Uganda. Reference tables for abnormal organometry were constructed. We identified risk factors for splenic enlargement, left liver lobe shrunkenness/enlargement and right liver lobe shrunkenness/enlargement. Detailed covariates on sociodemographics, water, sanitation and hygiene (WASH) conditions, treatment history and behavioural risk factors were measured in addition to the key exposures of *S. mansoni*, malaria, HIV and HBV. The main aim of this study was to assess the associations of current schistosome infections with abnormal organometry in the context of widespread MDA and co-endemic infections of malaria, HIV and HBV. A secondary aim was to construct height-standardised internal reference values from an infection-free population for hepatosplenic organometry and compare the utility of such a method with reference values provided by the WHO Niamey Protocol.

## Materials and methods

### Study design and participant sampling

This cross-sectional study was nested within the SchistoTrack cohort during the 2024 time point. SchistoTrack is a community-based prospective cohort established in 2022 across three rural districts in Uganda: Pakwach (along the Albert Nile), Buliisa (Lake Albert) and Mayuge (Lake Victoria).^24^ Households were selected by random sampling within villages. One adult (*≥*18 years) and one child (5–17 years) were selected by the household head for clinical assessment. Cohort details and sampling are further described by Puthur *et al*.^24^

### MDA history in study area

In these districts, annual school-based and community-based MDA with praziquantel was started in 2003 to target eligible individuals aged at least 5 years, except in 2019 (all districts) and 2022–2023 (Mayuge), when only school-based MDA was completed.^3,25^ As reported to the WHO Expanded Special Project for the Elimination of Neglected Tropical Diseases,^25^ of the 21 possible annual rounds of MDA before this study, Buliisa and Pakwach districts received only 14 rounds, whereas Mayuge district received 17 rounds. The most recent round of MDA in our study districts was in 2023 (Mayuge) and 2022 (Buliisa and Pakwach). The mean administrative treatment coverage for community-based MDA was 80.8% in Buliisa, 80.6% in Pakwach and 76.2% in Mayuge. Among all rounds of MDA implementation, Pakwach and Buliisa districts missed WHO-suggested targets of 75% treatment coverage by more than 10% for only 1 year, whereas Mayuge district missed this target by more than 10% for three non-consecutive years. All study districts had no MDA in 2007 and 2008.

### Organometry outcomes

Abdominal ultrasound examinations were performed within study villages where portable patient beds were brought by the study team and a private examination space was created by hanging privacy curtains within schools, churches, or other buildings used as a base for the SchistoTrack teams.^24^ The ultrasound machines and beds were sanitised between participants with ethanol. Examinations were conducted by sonographers previously trained on the WHO Niamey Protocol^21^ and retrained by SchistoTrack to improve standardisation.^26,27^ Philips Lumify C5-2 curved linear array transducers with Lenovo 8505-F tablets with Android 9 Pie were used in abdomen view. Participants were asked whether they had fasted, defined as no food or drink other than water for at least 2 hours before examination. Sonographers were blinded to all infection and clinical outcomes that were not assessed via ultrasound. Before field collection, inter-sonographer agreement for organ size was required to be within 2 cm during annual SchistoTrack training.

The left liver lobe was measured on a still image via a longitudinal section at the parasternal line with the abdominal aorta in view as a visual reference. For the measurement of the right liver, the start was at the mid-clavicular line when the interlobar fissure of the gallbladder was in view; the cranial margin was estimated behind the ribs from the start of the straight hepatic edge. Spleen lengths for normal rounded shapes were measured between the two poles, crossing through the splenic hilum. In case of distorted spleen shapes, the length was measured past the splenic hilum to avoid underestimation due to organ curvature. Each measurement was taken with on-screen callipers and saved as a labelled still image.

To define abnormalities and account for anthropometric variation, height-standardised reference values were constructed. Among all study participants, a healthy reference population of 786 individuals was identified. Participants had to (1) be negative for *S. mansoni*, malaria, HBV and HIV; (2) not be pregnant; (3) have reported no current (within the past 12 months) alcohol consumption and (4) have no liver fibrosis as diagnosed via ultrasound. Height deciles were constructed for this healthy population. Abnormal organometry for all study participants was identified in comparison to the number of SDs of organ size from the mean of organ size within the same height decile of the healthy reference population. Liver outcomes included five categories: severely shrunken (*≤ −*2 SD), moderately shrunken (*> −*2 to *≤ −*1 SD), normal (*−*1 to +1 SD), moderately enlarged (*≥* +1 to *<* +2 SD) and severely enlarged (*≥* +2 SD). Splenic outcomes included three categories: normal, moderately enlarged (*≥* +1 to *<* +2 SD) and severely enlarged (*≥* +2 SD).

### Exposures

Key exposures included *S. mansoni*, malaria, HBV and HIV. Stool samples were collected in labelled sample containers that were distributed to study participants by village health team members and were used to prepare double Kato–Katz smears from a single stool sample using 41.7 mg templates.^2,3,28^ Both slides were examined independently by two trained technicians for *S. mansoni* ova approximately 24 hours after preparation. A random 10% of slides per village were retained for rereading. Infection intensity was analysed as a continuous variable, entering the model via a natural logarithm transformation of eggs per gram (EPG) plus one. Finger-prick capillary blood was collected at the same village sites by trained technicians after cleaning the index finger with 70% ethanol and discarding the first drop, following the WHO guidelines for malaria diagnosis.^29^ Malaria (Bioline Malaria Ag P.f/Pan RDT, Abbott) and HBV (Determine HBsAg RDT, Abbott) tests were read after 15 min according to the manufacturer manual, as described in Lang *et al*.^30^ HIV testing followed the Uganda Ministry of Health national algorithm.^31^ The Determine HIV-1/2 (Abbott) RDT was used as the screening test, HIV 1/2 STAT-PAK (Chembio) as the confirmatory test for screen-positive results, and Bioline HIV 1/2 3.0 (Abbott) as the tie-breaker for cases where the first two tests disagreed, as described by Bui *et al*.^32^

Study nurses asked study participants whether they had received praziquantel within the past year from the village health teams outside of the study. If available from national control programmes, participants were treated yearly as part of SchistoTrack with praziquantel irrespective of their infection status. Food and water or soda were administered alongside the praziquantel by government healthcare nurses, and participants were monitored for any potential side effects. In 2024, praziquantel was administered through the study outreach. As praziquantel from the study outreach is provided after diagnosis, the administration of henceforth called study praziquantel is recorded and analysed from the preceding year. Participants who missed the preceding study time point (2023) were recorded as not treated by the study outreach. Detailed covariate definitions for sociodemographics, WASH and behavioural risk factors are described in online supplemental table S1 and were selected based on variables identified as relevant for hepatosplenic schistosomiasis, in particular PPF.^3^

### Statistical analysis

All statistical analyses were performed using R version 4.2.1.^33^ Multinomial logistic regression was used to estimate relative risk ratios (RRR) and 95% CIs for all outcomes with one regression each for the spleen, left liver lobe and right liver lobe. Children and adults were analysed in separate models to account for physiological differences in organ growth and risk profiles. Base models with key exposures *S. mansoni*, malaria, HIV and HBV, as well as age, sex and district, were constructed with HIV and HBV only in models for adults due to the limited prevalence (*<*5%) in children. Appropriateness of a multinomial model compared with a proportional-odds model was assessed with the Brant test.^34^ For variable selection, variables with less than 5% frequency in the study population were excluded. Candidate covariates were added individually to base models and tested using likelihood ratio tests; variables providing a significant improvement in model fit (p*<*0.05) were retained. To facilitate direct comparison of risk factors across different organs, the final multivariable models for each population (children and adults) consisted of the union of all significant variables identified for any of the three organ outcomes, respectively. Collinearity was assessed using generalised variance inflation factors implemented in the car package.^35,36^ Sensitivity analyses included collapsed outcome categories, cross-tabulation of left and right liver lobe classifications, and substitution of binary malaria RDT status with continuous log-transformed parasite density, as defined in Lang *et al*,^30^ in children’s spleen model. As a supplementary analysis, the models were refitted with directly observed study-administered praziquantel from the preceding year included alongside self-reported past-year praziquantel. *S. mansoni* EPG were additionally grouped into the four WHO intensity categories.^37^ Separately for children and for adults, a χ^2^ test assessed whether left liver lobe, right liver lobe, and spleen classifications differed across these categories.

Population attributable fractions (PAFs) were estimated using the G-computation framework^38^ to quantify the disease burden statistically attributable to each key infection. PAFs were estimated only for exposures that showed statistically significant associations (p*<*0.05) in the multivariable models. These values were derived by comparing the observed study population to hypothetical counterfactual scenarios. For each infection, a counterfactual population was constructed by setting the exposure for all participants to the reference level, zero EPG for *S. mansoni*, malaria-negative, HIV-negative or HBV-negative, while retaining observed values for all other covariates in the adjusted models. Uncertainty for these estimates was assessed via 95% CIs derived from 1000 bootstrap iterations.

To validate the construction of the internal reference population, we compared organometric classifications against the reference standards defined by the WHO Niamey Protocol.^21,39^

Participants were re-classified using the fixed height-dependent cut-offs provided in the Niamey guidelines (Annex C of the Niamey Protocol).^21^ We calculated the prevalence of abnormal organometry under both classification systems and assessed discordance to determine if external standards systematically overestimated or underestimated specific pathologies within the SchistoTrack cohort.

## Results

### Organometry outcomes and infection prevalence

A total of 3302 out of 3385 clinical participants underwent ultrasound examinations in 2024, with 3121 participants with complete data and the focus of analyses with 1507 children and 1614 adults (online supplemental figure S1). Participant characteristics are detailed in online supplemental tables S2 and S3. The median age was 39 years (IQR 30–50) for adults and 9 years (IQR 7–12) for children. The majority of adults were female (1051/1614; 65%), whereas the sex distribution was more balanced among children (670/1507; 44% female). Praziquantel uptake through MDA in the past year was low, reported by 20% (325/1614) of adults and 24% (358/1507) of children. Directly observed study-administered praziquantel at the preceding visit was recorded for 73.4% (2292/3121) of participants. Stratified by children and adults, 76.1% (1147/1507) of children and 70.9% (1145/1614) of adults received study-administered praziquantel. This coverage was 82.8% (1638/1978) among participants enrolled in 2022 and 91.7% (654/713) among those enrolled in 2023. Participants enrolled in 2024 had no preceding study visit and were recorded as untreated (0/430). Among the 2691 participants enrolled in 2022 or 2023, 399 were not seen at the preceding visit and were recorded as untreated (online supplemental table S2).

Organ dimensions derived from the healthy reference population are presented in online supplemental table S4. Abnormality prevalence varied by age group and organ (online supplemental table S2). Splenomegaly was common among children: moderate splenic enlargement was observed in 20% (297/1507) and severe enlargement in 9.4% (141/1507) of children. Moderate liver abnormalities affected approximately 10–13% of children, specifically moderate shrinkage (left lobe 12%, 182/1507; right lobe 13%, 195/1507) and moderate enlargement (left lobe 12%, 177/1507; right lobe 9.6%, 144/1507). Severe liver pathology was less frequent; severe shrunkenness was identified in 1.8% (27/1507) and 4.2% (63/1507) for the left and right lobes, respectively. Severe enlargement was found in 2.5% (37/1507) of left lobes and 0.6% (9/1507) of right lobes.

Among adults, splenic enlargement was also common, with 13% (216/1614) classified as moderately enlarged and 9.9% (160/1614) as severely enlarged. Moderate liver abnormalities were frequent; 9.7% (156/1614) and 14% (230/1614) had left and right lobe shrunkenness, respectively, while moderate enlargement was observed in 16% (254/1614) for the left lobe and 13% (209/1614) for the right lobe. Severe abnormalities were less common; severe shrunkenness affected 2.7% (43/1614) of left lobes and 4.5% (73/1614) of right lobes, while severe enlargement was identified in 5.1% (83/1614) of left lobes and 2.5% (40/1614) of right lobes. Differences in infection prevalence were observed between children and adults (online supplemental table S2 and online supplemental table S5). *S. mansoni* prevalence was significantly higher in children (58%, 878/1507) compared with adults (30%, 478/1614; χ^2^=259.1, p<0.001). Overall infection prevalence also varied by district (online supplemental table S6), as Pakwach had higher prevalence of *S. mansoni* and malaria than Mayuge, while HBV and HIV were modestly more common in Buliisa and Pakwach.

### Determinants of abnormal organometry for children

Multinomial models of abnormal organometry with selected covariates are presented in figures 1A,B, 2 and 3. For splenic morbidity in children, *S. mansoni* infection intensity was significantly associated with enlargement. For a doubling of *S. mansoni* egg counts, the relative risk of moderate enlargement increased by 4% and severe enlargement by 8% (per unit increase in log-transformed egg count: moderate enlargement RRR 1.06, 95% CI 1.01 to 1.12; severe enlargement RRR 1.12, 95% CI 1.04 to 1.20). Children with malaria had a 2.74-fold increase in the relative risk of moderate enlargement (95% CI 2.08 to 3.61) and a 3.96-fold increase in relative risk for severe enlargement (95% CI 2.64 to 5.92) compared with those without malaria. Additionally, the reported use of antimalarials in the past month was associated with a 51% increase in the risk of severe splenic enlargement (RRR 1.51; 95% CI 1.04 to 2.20).

**Figure 1:**
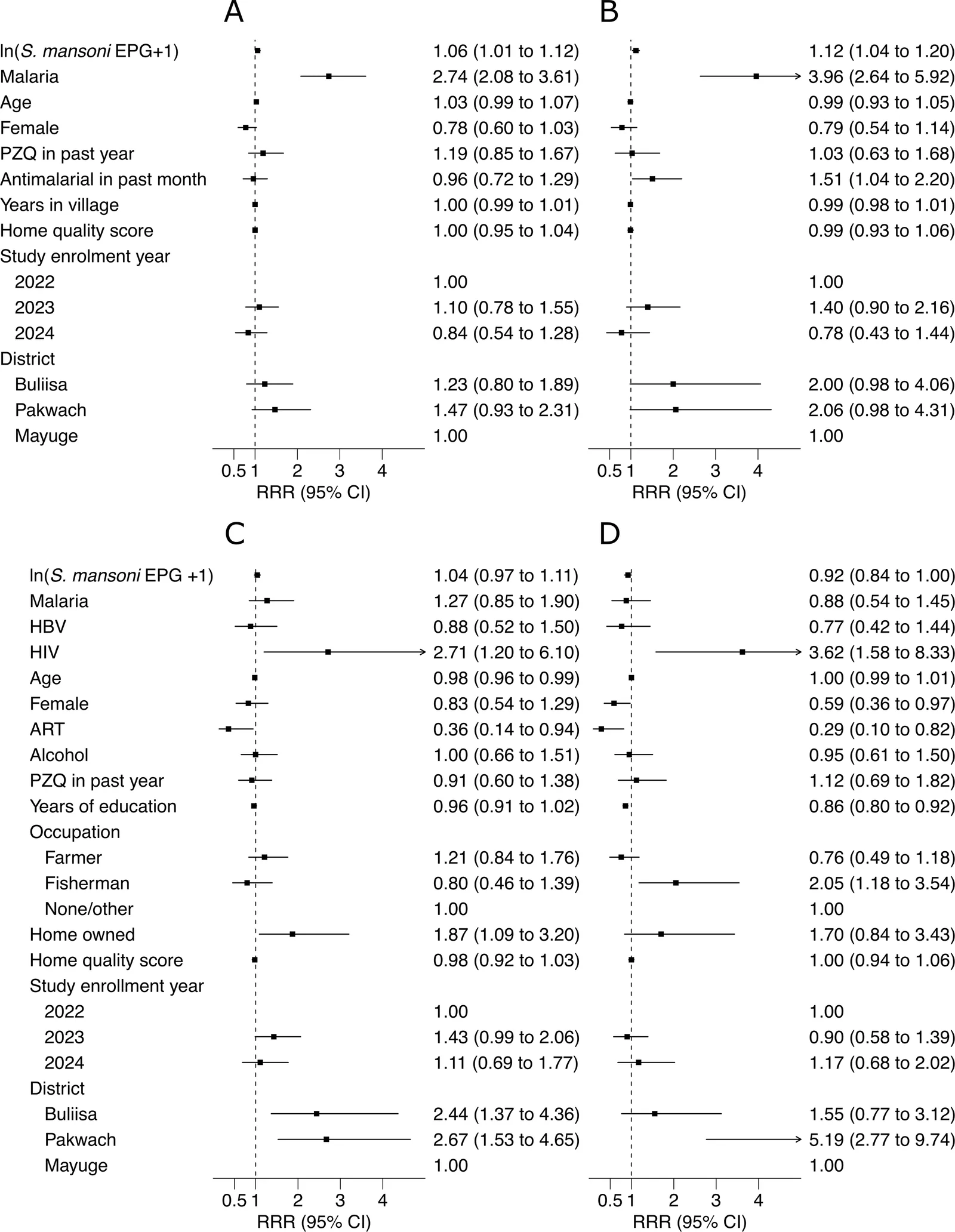
Determinants of spleen dimensions in children. Plots show RRRs with 95% CIs. (**A**) Children: Moderately enlarged. (**B**) Children: Severely enlarged. (**C**) Adults: Moderately enlarged. (**D**) Adults: Severely enlarged. GVIF was <5. ART, antiretroviral therapy; EPG, eggs per gram; GVIF, generalised variance inflation factor; HBV, hepatitis B virus; PZQ, praziquantel; RRR, relative risk ratio.

**Figure 2:**
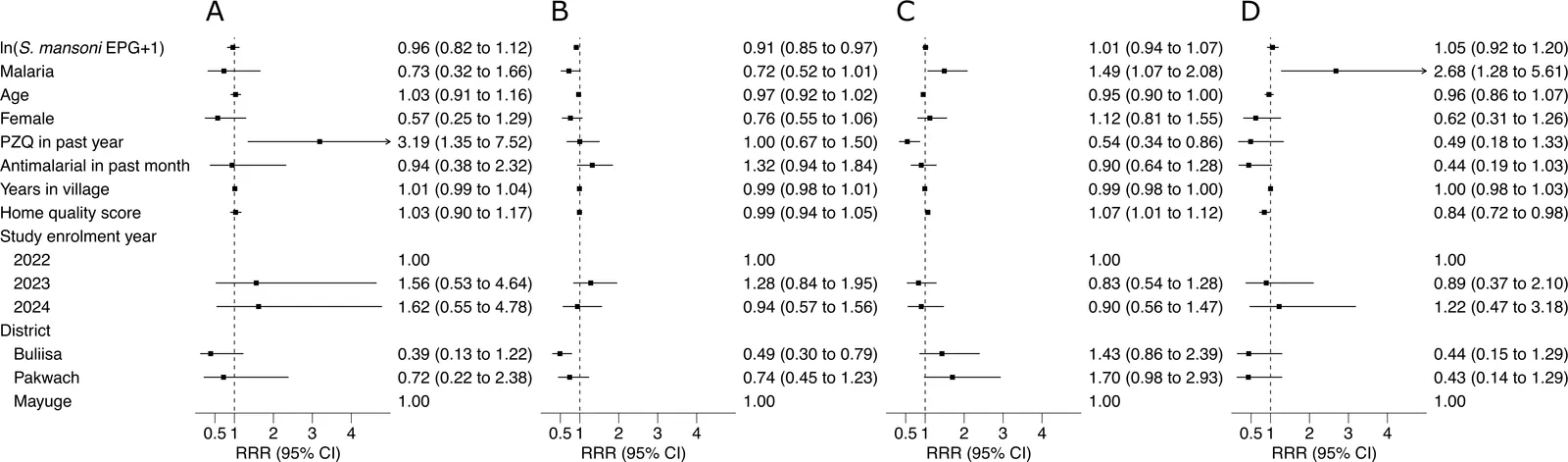
Determinants of left liver lobe abnormality in children. Plots show RRRs with 95% CIs. (**A**) Severely shrunken. (**B**) Moderately shrunken. (**C**) Moderately enlarged. (**D**) Severely enlarged. GVIF was <5. EPG, eggs per gram; GVIF, generalised variance inflation factor; PZQ, praziquantel; RRR, relative risk ratio.

**Figure 3:**
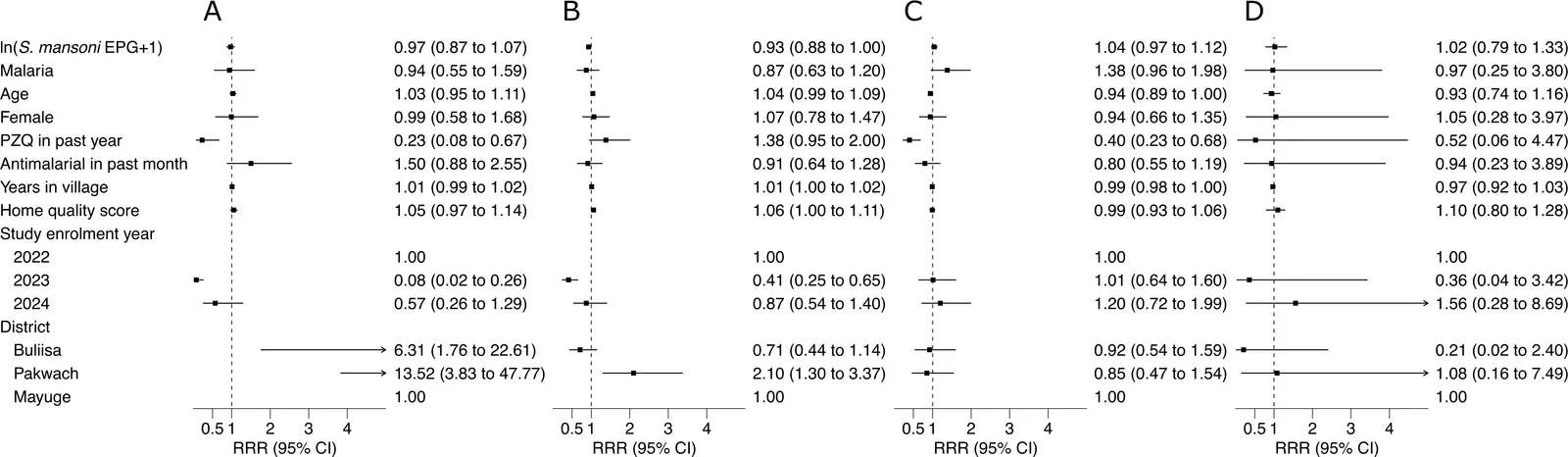
Determinants of right liver lobe dimensions in children. Plots show RRRs with 95% CIs. (**A**) Severely shrunken (**B**) Moderately shrunken (**C**) Moderately enlarged (**D**) Severely enlarged. GVIF was <5. EPG, eggs per gram; GVIF, generalised variance inflation factor; PZQ, praziquantel; RRR, relative risk ratio.

Regarding liver organometry in children, associations with *S. mansoni* intensity were unclear. Higher infection intensity was associated with a reduced risk of moderate left liver lobe shrinkage (RRR 0.91; 95% CI 0.85 to 0.97), but not severe left liver lobe shrinkage (RRR 0.96; 95% CI 0.82 to 1.12). Having malaria was associated with a 49% increase in the risk of moderate left lobe enlargement (RRR 1.49; 95% CI 1.07 to 2.08) and more than a twofold increase in the risk of severe enlargement (RRR 2.68; 95% CI 1.28 to 5.61). In children, WHO intensity categories did not capture differences in splenic or left liver lobe organometry, despite the associations of continuous infection intensity in these outcomes (online supplemental table S7).

Self-reported praziquantel treatment in the past year through MDA showed mixed associations with liver organometry. This praziquantel treatment history variable was identified as a significant correlate of severe left liver lobe shrinkage, associated with a 3.19-fold increase in relative risk (95% CI 1.35 to 7.52). Conversely, for the right liver lobe, treatment was associated with a 60% reduction in the risk of moderate enlargement (RRR 0.40; 95% CI 0.23 to 0.68). Study-administered observed praziquantel intake from the preceding year was analysed separately because it was not routine MDA and was associated with a lower relative risk of severe splenic enlargement in adults (RRR 0.53; 95% CI 0.33 to 0.83). In children specifically, it was associated with a lower relative risk of moderate right liver lobe enlargement (RRR 0.57; 95% CI 0.33 to 0.98), although this interval was wide and nearly included one. It was not associated with the other organometry classifications, including severe left liver lobe shrinkage (online supplemental figures S2–S7).

There were clear differences in hepatosplenic organometry between Western and Eastern Uganda. Living in Pakwach district was associated with a 2.10-fold increase in the relative risk of moderate shrinkage (95% CI 1.30 to 3.37) and a 13.52-fold increase for severe shrinkage (95% CI 3.83 to 47.77) as compared with living in Mayuge district. Similarly, residence in Buliisa increased the risk of severe right lobe shrinkage by over six times (RRR 6.31; 95% CI 1.76 to 22.61) as compared with Mayuge district.

### Determinants of abnormal organometry for adults

In adults, the determinants of organometry are shown in figures 1C,D, 4 and 5. For splenic morbidity, HIV infection was associated with a 2.71-fold increase in the relative risk of moderate enlargement (95% CI 1.20 to 6.10) and a 3.62-fold increase for severe enlargement (95% CI 1.58 to 8.33). Conversely, antiretroviral therapy (ART) use was associated with a 64% reduction in the relative risk of moderate enlargement (RRR 0.36; 95% CI 0.14 to 0.94) and a 71% reduction in the relative risk of severe enlargement (RRR 0.29; 95% CI 0.10 to 0.82). Demographic and geographic factors also contributed to splenic outcomes; occupation as a fisherman was associated with a 2-fold increase in the risk of severe enlargement (RRR 2.05; 95% CI 1.18 to 3.54) as compared with other or no occupations, while female sex was associated with lower relative risk of severe enlargement (RRR 0.59; 95% CI 0.36 to 0.97). Living in Pakwach district was strongly associated with a 5.19-fold increase in severe enlargement compared with living in Mayuge (95% CI 2.77 to 9.74).

**Figure 4:**
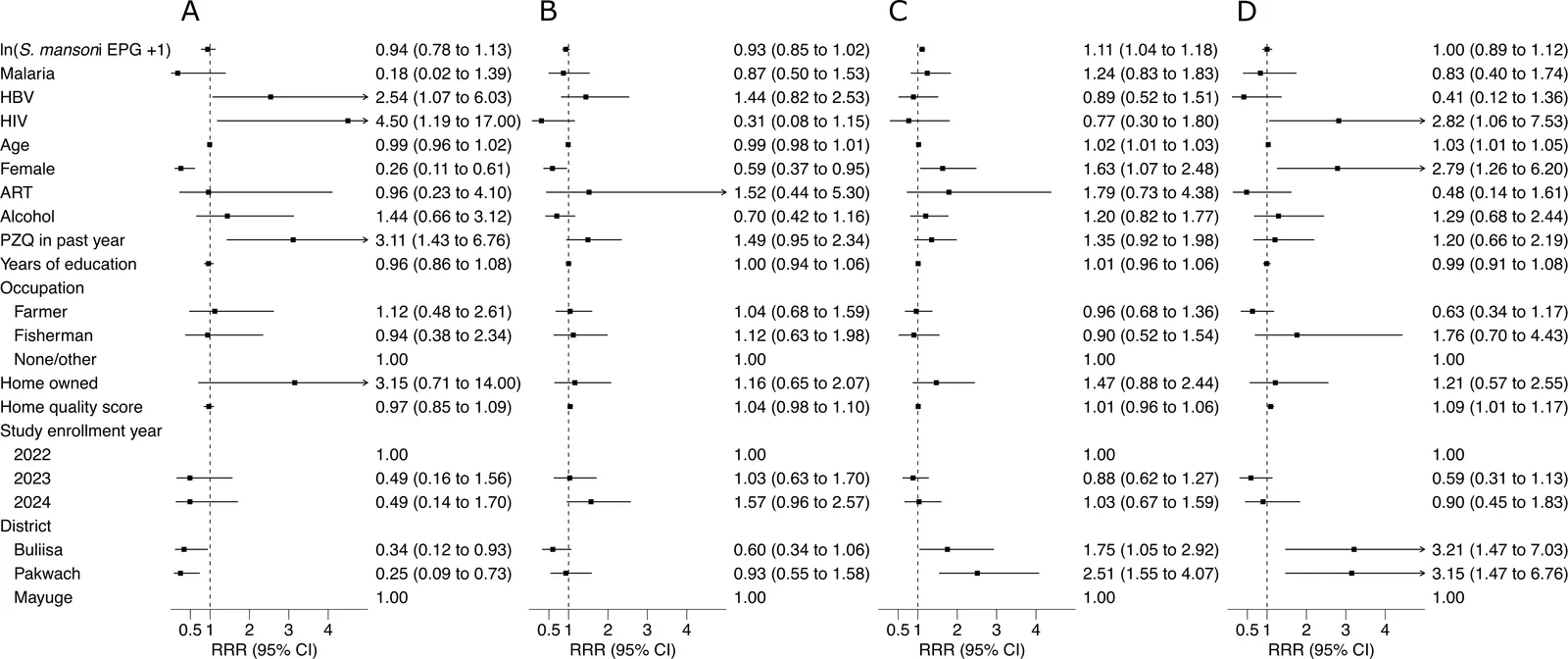
Determinants of left liver lobe dimensions in adults. Plots show RRRs with 95% CIs. (**A**) Severely shrunken. (**B**) Moderately shrunken. (**C**) Moderately enlarged. (**D**) Severely enlarged. GVIF remained within acceptable limits (*<*5). ART, antiretroviral therapy; EPG, eggs per gram; GVIF, generalised variance inflation factor; HBV, hepatitis B virus; PZQ, praziquantel; RRR, relative risk ratio.

**Figure 5:**
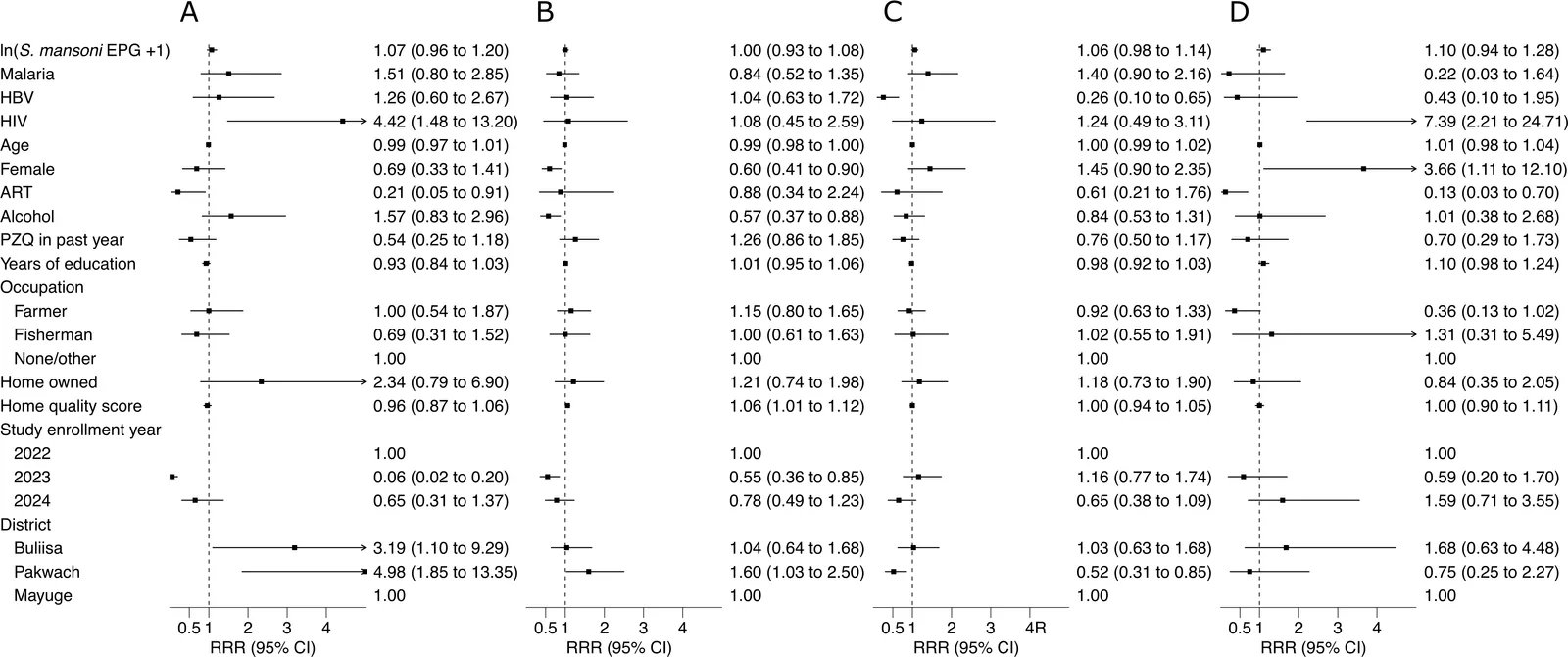
Determinants of right liver lobe dimensions in adults. Plots show RRRs with 95% CIs. (**A**) Severely shrunken. (**B**) Moderately shrunken. (**C**) Moderately enlarged. (**D**) Severely enlarged. GVIF was <5. ART, antiretroviral therapy; EPG, eggs per gram; GVIF, generalised variance inflation factor; HBV, hepatitis B virus; PZQ, praziquantel; RRR, relative risk ratio.

Regarding the left liver lobe, for a doubling of *S. mansoni* egg counts, the relative risk of moderate enlargement increased by 8% (per unit increase in log-transformed egg count: RRR 1.11; 95% CI 1.04 to 1.18). Viral infections were significant correlates of severe shrinkage, with HIV infection associated with a 4.50-fold increase in relative risk (95% CI 1.19 to 17.00) and HBV infection with a 2.54-fold increase in relative risk (95% CI 1.07 to 6.03). Praziquantel use in the past year from MDA was also associated with a 3.11-fold increase in the relative risk of severe shrinkage (95% CI 1.43 to 6.76). Female sex was a significant predictor for hepatomegaly, associated with increased relative risks for both moderate (RRR 1.63; 95% CI 1.07 to 2.48) and severe enlargement (RRR 2.79; 95% CI 1.26 to 6.20). Additionally, living in Pakwach or Buliisa was strongly associated with both moderate and severe left lobe enlargement as compared with living in Mayuge district. Even though continuous infection intensity was associated with the organometry outcomes for moderate enlargement, the WHO intensity categories did not capture differences in left liver lobe organometry (online supplemental table S7).

For the right liver lobe, HIV infection was strongly positively associated with increased relative risk of severe shrinkage (RRR 4.42, 95% CI 1.48 to 13.20) and severe enlargement (RRR 7.39, 95% CI 2.21 to 24.71). Similarly, for the left lobe, female sex was associated with a 3.66-fold increase in the risk of severe enlargement (95% CI 1.11 to 12.10). Similar to the left lobe, living in Pakwach corresponded to a nearly 5-fold increase (RRR 4.98; 95% CI 1.85 to 13.35) and living in Buliisa to a 3-fold increase (RRR 3.19; 95% CI 1.10 to 9.29) in relative risk of severe shrinkage when compared with living in Mayuge district.

### Population attributable fractions

PAFs for significant infection associations with organometry are presented in online supplemental table S8. In children, moderate (PAF 30.8%; 95% CI 21.2 to 41.0) and severe splenic enlargement (PAF 46.7%; 95% CI 31.7% to 61.3%) were often attributable to malaria. Nearly a quarter of cases of splenomegaly also were attributable to *S. mansoni* (severe enlargement PAF 23.6%; 95% CI 5.8% to 40.7%). Over 43% of severe left liver lobe enlargement was attributable to malaria (95% CI 13.9 to 69.9), while moderate enlargement was less attributable to malaria (PAF 17%; 95% CI 3.9% to 29.2%) of cases.

In adults, severe organometry outcomes were attributable to HIV. 16.4% of severe left liver lobe shrinkage was attributable to HIV (95% CI 3.1 to 29.2), with 8.9% (95% CI 0.5% to 17.6%) of severe right lobe shrinkage and 14.9% (95% CI 1.6% to 27.7%) of severe right lobe enlargement attributable to HIV.

*S. mansoni* accounted for 12.4% of moderate left liver lobe enlargement (95% CI 5.5% to 19.5%) of cases. Regarding the spleen, 7.2% of severe enlargement was attributable to HIV (95% CI 1.6 to 12.2).

### Sensitivity analyses and comparisons to WHO Niamey reference tables

Alternative models utilising collapsed outcome categories (shrunken, normal, enlarged) yielded results largely consistent with the primary adjusted models (online supplemental figures S8–S13). A sensitivity analysis substituting malaria status with continuous log-transformed parasite density reconfirmed the dose-dependent relationship between malaria and splenomegaly in children (online supplemental figure S14). For the outcome of hepatosplenomegaly, defined as any concurrent enlargement of both the liver and spleen, determinants mirrored those of the individual organs (online supplemental figures S15 and S16). We also present the frequency of infections and organometry outcomes by age in online supplemental figures S17–S20. The same models were refitted with study-administered praziquantel from the preceding year included alongside self-reported past-year praziquantel (online supplemental figures S2–S7). Study-administered praziquantel was associated with a lower relative risk of moderate right liver lobe enlargement in children (RRR 0.57; 95% CI 0.33 to 0.98) and of severe splenic enlargement in adults (RRR 0.53; 95% CI 0.33 to 0.83). It was not associated with other organometry classifications, including the severe left liver lobe shrinkage observed for self-reported past-year praziquantel. Self-reported past-year praziquantel and study-administered praziquantel at the preceding visit were not correlated (online supplemental table S9).

For both children and adults, the WHO Niamey reference standards systematically underestimated the prevalence of abnormal liver organometry while overestimating the prevalence of splenomegaly (online supplemental table S10). Using reference standards from the Niamey Protocol identified approximately half the prevalence of severe left liver lobe morbidity compared with SchistoTrack reference standards constructed here and classified only 0.1% of the population as having severe right liver lobe enlargement compared with 1.6% internally. Conversely, regarding the spleen, the Niamey Protocol references produced prevalence estimates for enlargement nearly double those of the internal reference (49.4% vs 26.0%).

## Discussion

Clinical guidance for hepatosplenic schistosomiasis is needed to enable morbidity surveillance and case management. We investigated the relationship between current *S. mansoni* infection intensity and abnormal organometry outcomes within the context of malaria, HIV and HBV co-endemic infection across 3121 children and adults within the SchistoTrack cohort. *S. mansoni* infections were associated with abnormal organometry in both children and adults after controlling for malaria, HIV and HBV co-endemic infections.

Despite severe splenomegaly in children being largely attributable to malaria (PAF 46.7%), *S. mansoni* infection intensity remained positively associated with moderate splenomegaly in children after controlling for malaria. Furthermore, 23.6% of severe splenomegaly was attributable to *S. mansoni* intensity. The WHO Niamey Protocol^21^ recommends excluding the assessment of splenomegaly for schistosomiasis classification in malaria-endemic areas. This guidance is not differentiated for children versus adults. Our study challenges this suggestion as schistosome infections remain associated with splenomegaly in children despite the co-endemicity of malaria. Given the high spatial overlap of schistosome and malaria infections and high prevalence of co-infections,^40–42^ following the current WHO Niamey protocol guidance^21^ would result in gross underestimations of schistosomiasis-related morbidities. Future revisions of the WHO Niamey Protocol should recommend that splenomegaly be assessed in malaria-endemic areas, while controlling for malaria co-infections in children only.

In children, only malaria was positively correlated to abnormal liver organometry, specifically for left liver lobe enlargement. This finding is notable given that the dominant malaria species in our study area is *Plasmodium falciparum*,^30^ which is not typically associated with chronic hepatic phase pathology in its life cycle. This contrasts with historical studies^43,44^ suggesting malarial hepatomegaly is common, though often transient. Given the low prevalence of severe liver enlargement in children (2.5%), this result should be interpreted with caution. Future research is needed to understand if hepatomegaly should be considered a clinical marker of chronic *P. falciparum* exposure in children.

Abnormal liver organometry was observed more often in adults than children with inconsistent associations with *S. mansoni* infection and clear associations with HIV and HBV. *S. mansoni* infection only showed a positive association with moderate left lobe enlargement. This association may be an indicator of early morbidity, as it has been suggested in Brazilian hospital-based studies that schistosome fibrosis and pathogenesis are first observed in the left liver lobe.^45–47^ This heterogeneity may be attributable to anatomical differences in blood flow from the portal vein, which differentially expose liver lobes to parasite eggs and gut-derived toxins.^47^ WHO intensity categories did not capture differences in organometry outcomes, despite the associations of continuous infection intensity with gradual increases in these outcomes.

There were associations of HIV and HBV with abnormal liver organometry. HBV was associated with a 2.54-fold higher RRR of severely shrunken left liver lobes. While HBV is a known cause of cirrhosis, it is unclear why this effect would be isolated to the left lobe, warranting further investigation. HIV was consistently associated with severe outcomes of both liver shrinkage and hepatomegaly in both the left and right liver lobes. HIV mono-infection is associated with diverse hepatic pathologies including fibrosis, fatty liver disease and opportunistic infections, and ART itself can contribute to hepatic pathology.^48–51^

Anjorin *et al*^3^ demonstrated that HIV infection significantly increased the likelihood of schistosomal PPF, independent of current *S. mansoni* infection status. Furthermore, Zhi *et al*^2^ identified HIV as a key driver of PPF and hepatosplenic multimorbidity using Bayesian multitask machine learning, reinforcing the hypothesis that viral co-infections accelerate the transition from inflammatory morbidity to chronic fibrosis for schistosomiasis-specific fibrosis patterns. While early schistosomal morbidity is believed to manifest in granulomatous-induced inflammation leading to hepatomegaly,^52,53^ advanced fibrosis related to PPF due to the condensed structure of the collagen matrix can result in reduced liver volume,^52,54^ perhaps even mimicking the atrophic outcome of viral cirrhosis though cirrhosis is assumed to be uncommon for hepatosplenic schistosomiasis.^8,54,55^ A recent meta-analysis^56^ found that co-infections with HBV increased the odds of cirrhosis when compared with singular infections, further supporting that viral-parasitic interactions (here with past schistosome infection) might be driving the liver pathology as opposed to singular infections. Our findings might be consistent with an interaction between HIV and past schistosome infection; however, it was not possible to assess this directly in this analysis. Future work is needed to assess HIV and schistosome interactions and ideally prospectively to know the sequence of exposures for untangling interactions from potential immune priming. Our findings underscore a critical gap in current MDA strategies, which typically focus on *S. mansoni* in isolation. HIV and HBV should be considered when developing morbidity assessment tools for schistosomiasis-related morbidity in adults.

We observed an association between self-reported praziquantel in the past year through MDA and severe liver shrinkage in adults and children. However, this result does not establish a clear negative effect of routine MDA on hepatosplenic organometry. The association may reflect reverse causality, where participants with established morbidity or symptoms are more likely to seek or be targeted for treatment, rather than a direct effect of the drug. Directly measured praziquantel receipt during community MDA in Mayuge has been about 50%.^57–59^ In this cohort, self-reported past-year praziquantel was 21.9%, whereas study-administered praziquantel from the preceding visit was 73.4%. We emphasise that this does not represent study adherence, because directly observed praziquantel intake was recorded at the preceding visit. Coverage was therefore below 100%, both because the participants recruited in 2024 had no preceding visit and because of cohort attrition.^24^

Directly observed, study-administered praziquantel showed no association with severe shrinkage of the left liver lobe; however, it was associated with a lower relative risk of severe splenic enlargement in adults (RRR 0.53). The reduced risk of splenic enlargement is relevant in people who may already have severe liver disease, because splenic enlargement can accompany obstructed hepatic blood flow arising from fibrosis and may mark a higher risk of decompensation.^60^ Regarding the association with moderate enlargement of the right liver lobe in children (RRR 0.57), caution is warranted, since the interval was wide and its upper bound fell only just below one.

The self-reported associations contrast with pre-MDA studies in children only suggesting praziquantel can reverse early hepatosplenic morbidity.^61^ Our results support recent analyses demonstrating persistent morbidity that is not amenable to preventive chemotherapy despite repeated MDA.^2,3,14^ Studies are currently underway within the SchistoTrack cohort to evaluate morbidity resolution prospectively.^24^

Spatial variation exists for the prevalence of abnormal organometry. Western districts (Pakwach and Buliisa) had the highest burden of severe liver shrinkage and splenic enlargement compared with the eastern district of Mayuge. Pakwach district specifically had the highest burden of abnormalities in both children and adults. Schistosome infection prevalence as well as prevalence for malaria, HBV and HIV varied by district, but would have been accounted for in our models and did not negate strong district effects. Future research is needed to determine whether unmeasured exposures distinct to the Albert Nile region or specific genetic factors are driving these disparities.

Methodologically, our study highlights limitations in the fixed height-bin approach currently employed by the Niamey Protocol,^21^ which relies on external reference values derived from a Senegalese population.^39^ The application of these external standards to our cohort resulted in systematic diagnostic error, likely driven by the limitations of the original reference dataset, which was generated in a pre-MDA context with a limited age range and exclusion criteria that did not account for HIV or HBV infections. By contrast, our internal references were derived from a larger, contemporary population with rigorous screening for co-endemic viruses and parasites, using height deciles to provide a more granular standardisation of organometry. Updated reference tables are needed to assess abnormal organometry. Future research should determine whether a global reference table, similar to WHO child growth standards, can be developed or if country-specific tables are necessary. The reference table generated in our study should be tested in other contexts; if generalisable, it could be used where local data are insufficient.

Strengths include a large age range, comprehensive risk factor assessment, robust models, PAFs and detailed reference tables. Limitations include the cross-sectional design, which precludes causal inference regarding the temporal sequence of exposures and organometry changes, and a focus on clinical interpretability rather than predictive modelling. In particular, associations with self-reported praziquantel from MDA in the past year should be interpreted carefully, as this variable may be susceptible to recall bias and misclassification if participants confuse praziquantel with other MDA campaigns. Prospective analyses are needed to assess the predictive performance of abnormal organometry for more severe schistosomiasis-related outcomes such as portal hypertension or variceal bleeding.

## Conclusions

Accurately defining routine clinical indicators of hepatosplenic schistosomiasis is necessary to enable the development of morbidity diagnosis and clinical management guidelines in schistosomiasis-endemic areas. *S. mansoni* remains correlated with splenomegaly despite co-endemic malaria, but as HIV appears associated with more severe hepatic organometry, future research must investigate potential interactions between these pathogens. Organometry and the reference table provided here should be considered in routine health system indicators for schistosomiasis-morbidity monitoring and large-scale surveillance surveys in SSA.

## Supplementary material

10.1136/bmjph-2026-005503 online supplemental file 1

10.1136/bmjph-2026-005503 online supplemental file 2

## Data Availability

No data are available.
