# Supplementary material for: Associations of current Schistosoma mansoni infections with hepatosplenic organometry: a community-based cross-sectional study within the SchistoTrack cohort in rural Uganda
Source: BMJ Public Health. 2026 Nov 9;4(4):e005503. doi: 10.1136/bmjph-2026-005503 (PMC13756384; doi:10.1136/bmjph-2026-005503)
Supplement: Supplementary file 1 [file bmjph-4-4-s001.docx]

Supporting information

**Supplementary Figures**

**Fig S1. Flowchart of study participant selection.** The diagram illustrates the derivation of the analytical sample (*n* = 3121) from the total clinical participants recruited in the SchistoTrack cohort (*n* = 3385) during January–February 2024. Exclusions were made for missing ultrasound examinations or incomplete covariate data. Participants may be missing more than one of the listed sources. The final population was stratified into children (aged 5–17 years; *n* = 1507) and adults (aged *≥*18 years; *n* = 1614).

**Fig S2. Determinants of left liver lobe dimensions in children, including study-administered praziquantel.** Plots show RRRs with 95% CIs. **A** Severely shrunken. **B** Moderately shrunken. **C** Moderately enlarged. **D** Severely enlarged.

**Fig S3. Determinants of right liver lobe dimensions in children, including study-administered praziquantel.** Plots show RRRs with 95% CIs. **A** Severely shrunken. **B** Moderately shrunken. **C** Moderately enlarged. **D** Severely enlarged.

**Fig S4. Determinants of spleen dimensions in children, including study-administered praziquantel.** Plots show RRRs with 95% CIs. **A** Moderately enlarged. **B** Severely enlarged.

**Fig S5. Determinants of left liver lobe dimensions in adults, including study-administered praziquantel.** Plots show RRRs with 95% CIs. **A** Severely shrunken. **B** Moderately shrunken. **C** Moderately enlarged. **D** Severely enlarged.

**Fig S6. Determinants of right liver lobe dimensions in adults, including study-administered praziquantel.** Plots show RRRs with 95% CIs. **A** Severely shrunken. **B** Moderately shrunken. **C** Moderately enlarged. **D** Severely enlarged.

**Fig S7. Determinants of spleen dimensions in adults, including study-administered praziquantel.** Plots show RRRs with 95% CIs. **A** Moderately enlarged. **B** Severely enlarged.

**Fig S8. Determinants of collapsed left liver lobe dimensions in children.** Plots show RRRs with 95% CIs. **A** Shrunken. **B** Enlarged.

**Fig S9. Determinants of collapsed right liver lobe dimensions in children.** Plots show RRRs with 95% CIs. **A** Shrunken. **B** Enlarged.

**Fig S10. Determinants of collapsed spleen dimensions in children.** Plots show ORs with 95% CIs.

**Fig S11. Determinants of collapsed left liver lobe dimensions in adults.** Plots show RRRs with 95% CIs. **A** Shrunken. **B** Enlarged.

**Fig S12. Determinants of collapsed right liver lobe dimensions in adults.** Plots show RRRs with 95% CIs. **A** Shrunken. **B** Enlarged.

**Fig S13. Determinants of collapsed spleen dimensions in adults.** Plots show ORs with 95% CIs.

**Fig S14. Determinants of spleen dimensions in children using continuous malaria parasite density.** The model substitutes binary malaria RDT status with natural log-transformed parasite density (ln(parasites*/µ*L + 1)). Plots show RRRs with 95% CIs. **A** Moderately enlarged. **B** Severely enlarged.

**Fig S15. Determinants of hepatosplenomegaly in children.** Plots show OR with 95% CIs for the composite outcome of concurrent liver and splenic enlargement.

**Fig S16. Determinants of hepatosplenomegaly in adults.** Plots show OR with 95% CIs for the composite outcome of concurrent liver and splenic enlargement.

**Fig S17. Frequency of infections by age.** Histograms display the age distribution of positive cases for each infection. **A** *S. mansoni*. **B** Malaria. **C** HBV. **D** HIV.

**Fig S18.	Frequency of left liver lobe outcomes by age. A** Moderately shrunken. **B**

Moderately enlarged. **C** Severely shrunken. **D** Severely enlarged.

**Fig S19. Frequency of right liver lobe outcomes by age. A** Moderately shrunken. **B**

Moderately enlarged. **C** Severely shrunken. **D** Severely enlarged.

**Fig S20. Frequency of spleen size outcomes by age. A** Moderately enlarged. **B** Severely enlarged.
