# Supplementary material for: Associations of current Schistosoma mansoni infections with hepatosplenic organometry: a community-based cross-sectional study within the SchistoTrack cohort in rural Uganda
Source: BMJ Public Health. 2026 Nov 9;4(4):e005503. doi: 10.1136/bmjph-2026-005503 (PMC13756384; doi:10.1136/bmjph-2026-005503)
Supplement: Supplementary file 2 [file bmjph-4-4-s002.docx]

# Supplementary Tables

**Table S1. Covariate definitions.** Detailed description of variable types and definitions for individual, household, and district level covariates. Abbreviations: ART (antiretroviral therapy).

## Variable Name Type Description

### Individual level

| **Age** | Continuous | Participant age to nearest year |
| --- | --- | --- |
| **Sex** | Binary | Participant sex  *Male = 0, Female = 1* |
| **Alcohol** | Binary | Self-reported alcohol use (ever or currently)  *No = 0, Yes = 1* |
| **Smoking** | Binary | Self-reported smoking (ever or currently)  *No = 0, Yes = 1* |
| **ART** | Binary | Self-reported ART use (ever or currently)  *No = 0, Yes = 1* |
| **Praziquantel in past year** | Binary | Self-reported treatment with praziquantel in past year  *No = 0, Yes = 1* |
| **Antimalarial in past month** | Binary | Self-reported antimalarial treatment in past month  *No = 0, Yes = 1* |
| **Education** | Continuous | Self-reported years of education attained |
| **Occupation** | Categorical | Self-reported occupation  *Fisherman, Farmer, None/Other (reference)* |
| **Water contact** | Binary | Self-reported participation in any activity involv-ing lake water contact  *No = 0, Yes = 1* |
| **Tribe** | Categorical | Self-reported participant tribe  *Alur, Musoga, Bagungu, Other (reference)* |
| **Majority religion** | Binary | Self-identified religion same as most common religion in village  *No = 0, Yes = 1* |

## Household level

**Enrollment year**	Continuous	Year household enrolled in study

Continued on next page

## Table S1 – continued from previous page

| **Variable Name** | **Type** | **Description** |
| --- | --- | --- |
| **Home quality** | Continuous | Sum of scores based on roof, floor, and wall ma-terial  *Floor: mud (+1), plastic (+2), wood (+3),*  *bricks/cement (+4)*  *Wall: mud/sticks (+1), plastic (+2), metal (+3),*  *bricks/cement (+4)*  *Roof: grass/papyrus (+1), sticks (+2), plastic*  *(+3), metal (+4)* |
| **Water purification** | Binary | Household uses any technique to make water safer to drink  *No = 0, Yes = 1* |
| **Hygiene** | Binary | Household has handwashing facility with soap and water  *No = 0, Yes = 1* |
| **Sanitation** | Binary | Household has improved sanitation (private pit/flush latrine)  *No = 0, Yes = 1* |
| **Household status** | Binary | Any member of household on local council, reli-gious/tribe leader, village health team, or beach management team  *No = 0, Yes = 1* |
| **Years in village** | Continuous | Number of years household head lived in village |
| **Own home** | Binary | Household head owns home  *No = 0, Yes = 1* |

## District level

**District**	Categorical	District household located in

*Buliisa, Pakwach, Mayuge (reference)*

**Table S2. Demographic, clinical, and parasitological characteristics of the study population.** Values are presented as n (%) for categorical variables and median (IQR) for continuous variables.

## Variable Adults Children

(N = 1614)	(N = 1507)

***S. mansoni*** (Positive)	478 (30%)	878 (58%)

**Malaria** (Positive)	211 (13%)	697 (46%)

**HBV** (Positive)	140 (8.7%)	22 (1.5%)

**HIV** (Positive)	138 (8.6%)	19 (1.3%)

## Left liver lobe

| Severely shrunken | 43 (2.7%) | 27 (1.8%) |
| --- | --- | --- |
| Moderately shrunken | 156 (9.7%) | 182 (12%) |
| Normal | 1078 (67%) | 1084 (72%) |
| Moderately enlarged | 254 (16%) | 177 (12%) |
|  |  | *Continued on next page...* |

**Table S2.** Table continued from previous page

Severely enlarged	83 (5.1%)	37 (2.5%)

## Right liver lobe

| Severely shrunken | 73 (4.5%) | 63 (4.2%) |
| --- | --- | --- |
| Moderately shrunken | 230 (14%) | 195 (13%) |
| Normal | 1062 (66%) | 1096 (73%) |
| Moderately enlarged | 209 (13%) | 144 (9.6%) |
| Severely enlarged | 40 (2.5%) | 9 (0.6%) |
| **Spleen** | | |
| Normal | 1238 (77%) | 1069 (71%) |
| Moderately enlarged | 216 (13%) | 297 (20%) |
| Severely enlarged | 160 (9.9%) | 141 (9.4%) |
| **Age (years)** | 39.0 (30.0, 50.0) | 9.0 (7.0, 12.0) |
| **Sex (Female)** | 1051 (65%) | 670 (44%) |
| **ART** | 110 (6.9%) | 13 (0.9%) |
| **Praziquantel in past year** | 325 (20%) | 358 (24%) |
| **Study praziquantel at preceding visit**^a^ | 1145 (71%) | 1147 (76%) |
| **Antimalarials** | 383 (24%) | 482 (32%) |
| **Alcohol intake** | 322 (20%) | - |
| **Smoking status** | 226 (14%) | - |
| **Years of education** | 4.0 (1.0, 6.0) | 2.0 (1.0, 3.0) |
| **Occupation** |  |  |
| None/other | 884 (55%) | - |
| Farmer | 510 (32%) | - |
| Fisherman | 220 (14%) | - |
| **Water contact** | 1393 (86%) | 1301 (86%) |
| **Tribe** |  |  |
| Alur | 992 (61%) | 944 (63%) |
| Bagungu | 147 (9.1%) | 150 (10.0%) |
| Musoga | 201 (12%) | 160 (11%) |
| Other | 274 (17%) | 253 (17%) |
| **Religion (majority)** | 1212 (75%) | 1154 (77%) |
| **Enrolment year** |  |  |
| 2022 | 1035 (64%) | 943 (63%) |
| 2023 | 363 (22%) | 350 (23%) |
| 2024 | 216 (13%) | 214 (14%) |
| **Home quality score** | 3.0 (3.0, 9.0) | 3.0 (3.0, 9.0) |
| **Water purification** | 361 (22%) | 339 (22%) |
| **Hygiene** | 133 (8.2%) | 129 (8.6%) |
| **Sanitation** | 924 (57%) | 866 (57%) |
| **Home ownership** | 1420 (88%) | 1322 (88%) |
| **Years in village** | 18.0 (8.0, 30.0) | 17.0 (8.0, 30.0) |
| **District** |  |  |
| Mayuge | 405 (25%) | 335 (22%) |
| Buliisa | 525 (33%) | 507 (34%) |

Pakwach	684 (42%)	665 (44%)

^a^ Study praziquantel at the preceding visit does not represent study adherence as it is impacted by both recruitment and study attrition

**Table S3. Lack of selection bias due to missing data.** Baseline characteristics of included vs. excluded participants. P-values calculated via Pearson’s Chi-squared test for categorical variables or Wilcoxon rank sum test for continuous variables.

**Variable	Excluded** (N = 181)	**Included** (N = 3121)	**p-value**

| *S. mansoni* | 77 (47%) | 1356 (43%) |  | 0*.*3 |
| --- | --- | --- | --- | --- |
| Malaria | 40 (24%) | 908 (29%) |  | 0*.*14 |
| HBV | 11 (6.5%) | 162 (5.2%) |  | 0*.*4 |
| HIV | 3 (2.5%) | 157 (5.0%) |  | 0*.*2 |
| Age | 16 (10, 35) | 21 (9, 40) |  | 0*.*3 |
| Sex (female) | 96 (53%) | 1721 (55%) |  | 0*.*6 |
| ART | 2 (1.2%) | 123 (4.0%) |  | 0*.*075 |
| Praziquantel in past year | 20 (15%) | 683 (22%) |  | 0*.*066 |
| Antimalarials in past month | 46 (35%) | 865 (28%) |  | 0*.*074 |
| Alcohol | 16 (12%) | 331 (14%) |  | 0*.*4 |
| Smoking | 9 (6.6%) | 230 (10.0%) |  | 0*.*2 |
| Years of education | 3 (1, 5) | 3 (1, 5) |  | 0*.*6 |
| Occupation |  |  |  | 0*.*4 |
| Farmer | 24 (13%) | 528 (17%) |  |  |
| Fisherman | 15 (8.3%) | 221 (7.1%) |  |  |
| None/Other | 142 (78%) | 2372 (76%) |  |  |
| Water contact | 154 (85%) | 2694 (86%) |  | 0*.*6 |
| Tribe |  |  |  | 0*.*8 |
| Alur | 118 (65%) | 1936 (62%) |  |  |
| Bagungu | 16 (8.8%) | 297 (9.5%) |  |  |
| Musoga | 17 (9.4%) | 361 (12%) |  |  |
| Other | 30 (17%) | 527 (17%) |  |  |
| Majority religion | 138 (76%) | 2366 (76%) |  | 0*.*9 |
| Enrolment year |  |  |  | 0*.*3 |
| 2022 | 106 (59%) | 1978 (63%) |  |  |
| 2023 | 43 (24%) | 713 (23%) |  |  |
| 2024 | 32 (18%) | 430 (14%) |  |  |
| Home quality | 3 (3, 9) | 3 (3, 9) |  | 0*.*8 |
| Water purification | 43 (24%) | 700 (22%) |  | 0*.*7 |
| Hygiene | 19 (10%) | 262 (8.4%) |  | 0*.*3 |
| Sanitation | 103 (57%) | 790 (57%) | *>* | 0*.*9 |
| Household status | 70 (39%) | 1123 (36%) |  | 0*.*5 |
| Years in village | 20 (9, 33) | 18 (8, 30) |  | 0*.*3 |
| Home ownership | 144 (80%) | 2742 (88%) |  | 0*.*001 |
| District |  |  |  | 0*.*5 |
| Buliisa | 64 (35%) | 1032 (33%) |  |  |
| Mayuge | 36 (20%) | 740 (24%) |  |  |
| Pakwach | 81 (45%) | 1349 (43%) |  |  |

**Table S4. Combined reference values for liver and spleen size classes by height.** All measurements are in cm. Values denote the range inclusive of the boundaries. Abbreviations: Sev, Severe abnormality (*>* 2 SD or *< −*2 SD); Mod, Moderate abnormality (1 to 2 SD or *−*1 to *−*2 SD); Enlg, Enlarged; Shrunk, Shrunken.

**Height n**

**Left liver lobe	Right liver lobe	Spleen**

**Sev	Mod	Normal	Mod	Sev	Sev	Mod	Normal	Mod	Sev	Mod	Sev**

(cm)	*Shrunk Shrunk	Enlg	Enlg Shrunk Shrunk	Enlg	Enlg	Enlg	Enlg*

| 75–122 | 82 | *<*4.77 | 4.77–5.85 | 5.85–8.02 | 8.02–9.10 | *>*9.10 | *<*5.32 | 5.32–6.51 | 6.51–8.90 | 8.90–10.09 | *>*10.09 | 11.06–12.95 | *>*12.95 |
| --- | --- | --- | --- | --- | --- | --- | --- | --- | --- | --- | --- | --- | --- |
| 123–136 | 78 | *<*4.92 | 4.92–5.99 | 5.99–8.14 | 8.14–9.21 | *>*9.21 | *<*5.22 | 5.22–6.66 | 6.66–9.54 | 9.54–10.98 | *>*10.98 | 11.00–12.41 | *>*12.41 |
| 137–144 | 80 | *<*5.25 | 5.25–6.75 | 6.75–9.75 | 9.75–11.25 | *>*11.25 | *<*5.43 | 5.43–7.29 | 7.29–11.02 | 11.02–12.88 | *>*12.88 | 13.31–15.72 | *>*15.72 |
| 145–150 | 76 | *<*5.15 | 5.15–6.79 | 6.79–10.08 | 10.08–11.72 | *>*11.72 | *<*5.61 | 5.61–7.56 | 7.56–11.46 | 11.46–13.41 | *>*13.41 | 12.70–14.97 | *>*14.97 |
| 151–155 | 80 | *<*5.25 | 5.25–6.74 | 6.74–9.71 9.71–11.19 *>*11.19 | | | *<*6.53 | 6.53–8.25 | 8.25–11.71 11.71–13.44 | | *>*13.44 | 12.47–14.80 | *>*14.80 |
| 156–159 | 86 | *<*5.21 | 5.21–6.91 | 6.91–10.33 10.33–12.04 *>*12.04 | | | *<*6.38 | 6.38–8.13 | 8.13–11.62 11.62–13.37 | | *>*13.37 | 13.81–16.54 | *>*16.54 |
| 160–162 | 78 | *<*4.66 | 4.66–6.42 | 6.42–9.92 | 9.92–11.68 | *>*11.68 | *<*6.35 | 6.35–8.18 | 8.18–11.83 11.83–13.65 | | *>*13.65 | 13.22–15.47 | *>*15.47 |
| 163–166 | 86 | *<*5.19 | 5.19–6.78 | 6.78–9.95 | 9.95–11.54 | *>*11.54 | *<*6.29 | 6.29–8.11 | 8.11–11.75 11.75–13.57 | | *>*13.57 | 13.41–15.74 | *>*15.74 |
| 167–170 | 63 | *<*4.42 | 4.42–6.20 | 6.20–9.74 | 9.74–11.51 | *>*11.51 | *<*6.19 | 6.19–7.97 | 7.97–11.52 11.52–13.29 | | *>*13.29 | 12.99–15.14 | *>*15.14 |
| 171–190 | 77 | *<*5.02 | 5.02–6.66 | 6.66–9.94 | 9.94–11.57 | *>*11.57 | *<*6.54 | 6.54–8.03 | 8.03–11.83 11.83–13.59 | | *>*13.59 | 14.03–16.47 | *>*16.47 |

**Table S5. Prevalence of co-infections.** Counts and percentages of participants with specific co-infection pairs stratified by age group.

| **Co-infection** | **Children (n = 1507)** | **Adults (n = 1614)** |
| --- | --- | --- |
| *S. mansoni* - malaria | 437 (29 %) | 74 (5%) |
| *S. mansoni* - HBV | 17 (1%) | 57 (4%) |
| *S. mansoni* - HIV | 14 (1%) | 37 (2%) |
| Malaria - HBV | 8 (1%) | 18 (1%) |
| Malaria - HIV | 6 (*<*1%) | 18 (1%) |
| HBV - HIV | 0 (0%) | 13 (1%) |

**Table S6.** Prevalence of Infections by District

| **District** | **Total N** | ***S. mansoni*** | **Malaria** | **HBV** | **HIV** |
| --- | --- | --- | --- | --- | --- |
| Buliisa | 1032 | 483 (46.8%) | 268 (26.0%) | 61 (5.9%) | 54 (5.2%) |
| Mayuge | 740 | 164 (22.2%) | 130 (17.6%) | 21 (2.8%) | 31 (4.2%) |
| Pakwach | 1349 | 709 (52.6%) | 510 (37.8%) | 80 (5.9%) | 72 (5.3%) |

**Table S7. Organometry classifications by WHO *S. mansoni* intensity category.** Cells are n/N (%), where N is the number of participants in that age group and WHO category. Categories are negative (0 EPG), light (1–99 EPG), moderate (100–399 EPG), and heavy (*≥*400 EPG). The p-value is from a chi-squared test of organometry classification by WHO category.

***Children***

**Left liver lobe**

Negative 0 EPG

Light 1–99 EPG

Moderate 100–399 EPG

Heavy

*≥*400 EPG

*p*-value

| Severely shrunken | 14/629 (2.2%) | 8/385 (2.1%) | 1/278 (0.4%) | 4/215 (1.9%) |  |
| --- | --- | --- | --- | --- | --- |
| Moderately shrunken | 99/629 (15.7%) | 47/385 (12.2%) | 19/278 (6.8%) | 17/215 (7.9%) |  |
| Normal | 434/629 (69.0%) | 278/385 (72.2%) | 214/278 (77.0%) | 158/215 (73.5%) | 0.004 |
| Moderately enlarged | 72/629 (11.4%) | 38/385 (9.9%) | 37/278 (13.3%) | 30/215 (14.0%) |  |
| Severely enlarged | 10/629 (1.6%) | 14/385 (3.6%) | 7/278 (2.5%) | 6/215 (2.8%) |  |

Continued on next page

**Table S7 – continued from previous page**

|  | Negative 0 EPG | Light 1–99 EPG | Moderate 100–399 EPG | Heavy  *≥*400 EPG | *p*-value |
| --- | --- | --- | --- | --- | --- |
| **Right liver lobe**  Severely shrunken | 19/629 (3.0%) | 19/385 (4.9%) | 16/278 (5.8%) | 9/215 (4.2%) |  |
| Moderately shrunken | 99/629 (15.7%) | 34/385 (8.8%) | 35/278 (12.6%) | 27/215 (12.6%) |  |
| Normal | 457/629 (72.7%) | 291/385 (75.6%) | 198/278 (71.2%) | 150/215 (69.8%) | 0.059 |
| Moderately enlarged | 50/629 (7.9%) | 40/385 (10.4%) | 26/278 (9.4%) | 28/215 (13.0%) |  |
| Severely enlarged | 4/629 (0.6%) | 1/385 (0.3%) | 3/278 (1.1%) | 1/215 (0.5%) |  |
| **Spleen**  Normal | 487/629 (77.4%) | 260/385 (67.5%) | 188/278 (67.6%) | 134/215 (62.3%) |  |
| Moderately enlarged | 104/629 (16.5%) | 80/385 (20.8%) | 57/278 (20.5%) | 56/215 (26.0%) | *<* 0*.*001 |
| Severely enlarged | 38/629 (6.0%) | 45/385 (11.7%) | 33/278 (11.9%) | 25/215 (11.6%) |  |
| ***Adults*** |  |  |  |  |  |
| **Left liver lobe**  Severely shrunken | 34/1136 (3.0%) | 5/276 (1.8%) | 3/115 (2.6%) | 1/87 (1.1%) |  |
| Moderately shrunken | 116/1136 (10.2%) | 22/276 (8.0%) | 15/115 (13.0%) | 3/87 (3.4%) |  |
| Normal | 762/1136 (67.1%) | 190/276 (68.8%) | 71/115 (61.7%) | 55/87 (63.2%) | 0.060 |
| Moderately enlarged | 161/1136 (14.2%) | 48/276 (17.4%) | 23/115 (20.0%) | 22/87 (25.3%) |  |
| Severely enlarged | 63/1136 (5.5%) | 11/276 (4.0%) | 3/115 (2.6%) | 6/87 (6.9%) |  |
| **Right liver lobe**  Severely shrunken | 40/1136 (3.5%) | 20/276 (7.2%) | 6/115 (5.2%) | 7/87 (8.0%) |  |
| Moderately shrunken | 160/1136 (14.1%) | 37/276 (13.4%) | 22/115 (19.1%) | 11/87 (12.6%) |  |
| Normal | 761/1136 (67.0%) | 172/276 (62.3%) | 73/115 (63.5%) | 56/87 (64.4%) | 0.011 |
| Moderately enlarged | 152/1136 (13.4%) | 32/276 (11.6%) | 13/115 (11.3%) | 12/87 (13.8%) |  |
| Severely enlarged | 23/1136 (2.0%) | 15/276 (5.4%) | 1/115 (0.9%) | 1/87 (1.1%) |  |
| **Spleen**  Normal | 884/1136 (77.8%) | 208/276 (75.4%) | 85/115 (73.9%) | 61/87 (70.1%) |  |
| Moderately enlarged | 136/1136 (12.0%) | 44/276 (15.9%) | 18/115 (15.7%) | 18/87 (20.7%) | 0.210 |
| Severely enlarged | 116/1136 (10.2%) | 24/276 (8.7%) | 12/115 (10.4%) | 8/87 (9.2%) |  |

**Table S8. Estimated Population Attributable Fraction in organometry by infection.** Values represent the estimated proportion of organ abnormalities statistically attributable to each specific infection using G-computation. All reported values correspond to exposures with significant coefficients (*p <* 0*.*05) in multivariable models. 95% CIs were derived from bootstrap resampling (n=1000). Positive values indicate the percentage of the outcome prevalence that is statistically attributable to the infection (i.e., the expected reduction in prevalence if the infection were removed). Negative values indicate that the infection is associated with a lower percentage of that specific category.

|  | | **Shrunken**  (% attributable change [95% CI]) |  | **Enlarged**  (% attributable change [95% CI]) |  |
| --- | --- | --- | --- | --- | --- |
| **Exposure** | **Organ** | Moderate Severe |  | Moderate Severe |  |
| ***Children*** |  |  |  |  |  |
| **Malaria** | Left Liver | -15.5 (-28.1, -2.9) – |  | 17.0 (3.9, 29.2) 43.0 (13.9, 69.9) |  |
|  | Spleen | – – |  | 30.8 (21.2, 41.0) 46.7 (31.7, 61.3) |  |
| ***S. mansoni* EPG+1** | Spleen | – – |  | – 23.6 (5.8, 40.7) |  |
| ***Adults*** |  |  |  |  |  |
| ***S. mansoni* EPG+1** | Left Liver | -10.1 (-19.2, -0.7) – |  | 12.4 (5.5, 19.5) – |  |
|  | Spleen | – – |  | – -11.2 (-22.2, -1.5) |  |
| **HIV** | Left Liver | -6.9 (-17.8, -1.0) 16.4 (3.1, 29.2) |  | – 9.4 (0.4, 17.7) |  |
|  | Right Liver | – 8.9 (0.5, 17.6) |  | – 14.9 (1.6, 27.7) |  |
|  | Spleen | – – |  | 4.9 (0.2, 9.2) 7.2 (1.6, 12.2) |  |

**Table S9. Self-reported past-year praziquantel and study-administered praziquantel at the preceding visit.** Cells are n (row %) with Phi (*ϕ*) coefficient = 0.03 (95% CI *−*0*.*01 to 0.06).

Study praziquantel at the preceding visit

| Self-reported past-year praziquantel | No | Yes | Total |
| --- | --- | --- | --- |
| No | 663 (27.2%) | 1775 (72.8%) | 2438 |
| Yes | 166 (24.3%) | 517 (75.7%) | 683 |
| Total | 829 | 2292 | 3121 |

## Table S10. Comparison of organometric prevalence using Internal (SchistoTrack) vs. WHO/Niamey protocol reference standards [21].

**Internal	WHO (Niamey)**

| **Organ / Classification** | **n** | **%** |  | **n** | **%** |  |
| --- | --- | --- | --- | --- | --- | --- |
| **Right liver lobe**  Severely Shrunken | 136 | 4.4 |  | 97 | 3.1 |  |
| Moderately Shrunken | 425 | 13.6 |  | 520 | 16.7 |  |
| Normal | 2158 | 69.1 |  | 2388 | 76.5 |  |
| Moderately Enlarged | 353 | 11.3 |  | 112 | 3.6 |  |
| Severely Enlarged | 49 | 1.6 |  | 4 | 0.1 |  |
| **Left liver lobe** | | | | | |  |
| Severely Shrunken | 70 | 2.2 |  | 36 | 1.2 |  |
| Moderately Shrunken | 338 | 10.8 |  | 318 | 10.2 |  |
| Normal (0) | 2162 | 69.3 |  | 2421 | 77.6 |  |
| Moderately Enlarged | 431 | 13.8 |  | 289 | 9.3 |  |
| Severely Enlarged | 120 | 3.8 |  | 57 | 1.8 |  |
| **Spleen** | | | | | |  |
| Severely Shrunken | – | – |  | 9 | 0.3 |  |
| Moderately Shrunken | – | – |  | 26 | 0.8 |  |
| Normal | 2307 | 73.9 |  | 1542 | 49.4 |  |
| Moderately Enlarged | 513 | 16.4 |  | 903 | 28.9 |  |
| Severely Enlarged | 301 | 9.6 |  | 641 | 20.5 |  |
